# ﻿A new species of *Chanohirata* (Hemiptera, Cicadellidae, Deltocephalinae, Penthimiini) from southern China with its complete genome

**DOI:** 10.3897/zookeys.1259.163605

**Published:** 2025-11-12

**Authors:** Mingming Sun, Tielong Xu, Guy Smagghe, Renhuai Dai, Xianyi Wang, Jiajia Wang

**Affiliations:** 1 Institute of Entomology, Guizhou University, The Provincial Key Laboratory for Agricultural Pest Management Mountainous Region, Guiyang, Guizhou 550025, China Guizhou University Guiyang China; 2 Guizhou Provincial Engineering Research Center of Medical Resourceful Healthcare Products, Guiyang Healthcare Vocational University, Guiyang, Guizhou, China Guiyang Healthcare Vocational University Guiyang China; 3 Department of Plants and Crops, Ghent University, Ghent 9000, Belgium Ghent University Ghent Belgium; 4 Department of Biology, Vrije Universiteit Brussels (VUB), 1050 Brussels, Belgium Vrije Universiteit Brussels (VUB) Brussels Belgium; 5 Engineering Research Center of Medical Biotechnology, School of Biology and Engineering, Guizhou Medical University, Guiyang, Guizhou, China Guizhou Medical University Guiyang China; 6 College of Biology and Food Engineering, Chuzhou University, Chuzhou, Anhui, China Chuzhou University Chuzhou China

**Keywords:** Checklist, mitogenome, morphology, phylogenetic, taxonomy

## Abstract

The genus *Chanohirata* Hayashi & Machida, 1996 is the second most speciose genus within the tribe Penthimiini, after *Penthimia* Germar, 1821 and is nearly endemic to China. This study provides the description of a new species, *Chanohirata pala* Sun, Xu & Dai, **sp. nov.**, discovered in Yunnan, southern China and a phylogenetic analysis including this and related taxa. The new species is diagnostically distinguished by its broad, shovel-shaped aedeagal base and a body length of 4.0–4.2 mm, with characteristic yellowish-brown coloration. A novel identification key is presented for all known *Chanohirata* species, based primarily on male aedeagal morphology, accompanied by a detailed geographic distribution map. Given the high morphological similarity among species within the genus, we complemented traditional taxonomy with molecular data by sequencing and assembling the complete mitochondrial genome of *C. pala* Sun, Xu & Dai, **sp. nov.** The mitogenome is 15,433 bp in length (GenBank accession number: PQ615145) and was analyzed for genome organization, base composition, relative synonymous codon usage, amino acid frequency, tRNA secondary structures, and phylogenetic relationships. Currently, *Chanohirata* comprises 15 species globally. Phylogenetic analyses incorporating mitochondrial protein-coding genes (PCGs), 12S rRNA and 16S rRNA, confirm that *C. pala* Sun, Xu & Dai, **sp. nov.** clusters firmly within the Deltocephalinae subfamily and is nested in the tribe Penthimiini. Notably, *C. pala* Sun, Xu & Dai, **sp. nov.** forms a sister clade with *C. theae* (Matsumura, 1912) and *C. hamata* (Wang & Zhang, 2019). This integrative approach combining detailed morphological examination with complete mitogenome sequencing advances our understanding of species boundaries and evolutionary history within *Chanohirata*, providing a valuable framework for future taxonomic and phylogenetic studies in Penthimiini.

## ﻿Introduction

Penthimiini was established by Kirschbaum (1868), it is one of the 39 tribes within the subfamily Deltocephalinae, comprising approximately 217 species across 46 genera, with a global zoogeographic distribution (Zahniser and Dietrich 2013; Wang and Zhang 2022, 2024) and it is hypothesized that the tribe Penthimiini originated in the Oriental region (Evans 1972; Cao et al. 2022), and *Chanohirata* most probably originated in southern China at 35.41 Ma during the late Eocene (Wang et al. 2024a), the leafhopper genus *Chanohirata*, established by Hayashi and Machida (1996) with *Penthimia theae* Matsumura, 1912 as the type species, initially included only this single species. Subsequent taxonomic and phylogenetic research has expanded the genus to 15 known species worldwide (Cheng and Li 2005; Fu and Zhang 2015; Xu and Dai 2020; Xu et al. 2020a; Wang and Zhang 2019, 2022; Wang et al. 2024a), for which we now provide an updated checklist and identification key.

Mitochondrial genome (mitogenome) data have emerged as powerful tools for resolving species identification and clarifying phylogenetic relationships due to their structural and compositional stability, maternal inheritance, and limited recombination (Simon et al. 2006; Cameron 2014b; Guo et al. 2024; Wang et al. 2024b; Lu et al. 2024). Typically, insect mitogenomes range from 15 to 20 kb, comprising 22 tRNAs, 13 protein-coding genes (PCGs), two ribosomal RNA genes, and a control region (Clary and Wolstenholme 1985; Cheng et al. 2000).

Within *Chanohirata*, morphological similarity, particularly in external features and male genitalia, poses significant challenges for species delimitation, making morphological identification alone often insufficient, for example, Viraktamath et al. (2012) misidentified *Japanagallia hamata* as *Japanagallia neohamata* due to its very similar color and form (Li et al. 2014) and species identification for taxa not reliably identified using *COX1* or *CYTB* might be best addressed through use of multiple mitochondrial DNA fragments or other newly developed markers (Viricel and Rosel 2012). This highlights a critical research gap and the need for integrative taxonomy, combining molecular and morphological data to enhance species identification accuracy. However, only four complete mitogenomes of Penthimiini species are available in GenBank: *C. hamata* (Wang and Zhang 2019) (NC051985; Xu and Dai 2020), *C. theae* (Matsumura, 1912) (NC081953; Lv 2023), *Penthimia melanocephala* Motschulsky, 1863 (MT768010; Xu 2020), and *Penthimia* sp. (PP856705; Hassan and Xing 2025). This limited mitogenomic representation hampers comprehensive phylogenetic analyses and evolutionary studies within the genus and tribe. Expanding mitogenomic datasets is therefore essential to improve molecular taxonomy and deepen understanding of Penthimiini and Deltocephalinae evolutionary history.

In this study, we describe a new species, *Chanohirata pala* sp. nov., based on specimens collected from Yunnan, southern China. We provide the complete mitochondrial genome sequence of this species, enhancing the molecular resources for the genus. Furthermore, we offer an updated checklist and identification key for all 16 known male *Chanohirata* species and investigate the phylogenetic placement of *C. pala* sp. nov. within Deltocephalinae using mitogenomic data. This integrative approach addresses the taxonomic challenges posed by morphological similarity and contributes to resolving the evolutionary relationships within this diverse leafhopper group.

## ﻿Material and methods

### ﻿Taxon sampling and DNA extraction

Specimens used in this study were collected using a sweep net and are deposited at the Institute of Entomology, Guizhou University, Guiyang, China (**GUGC**). External morphological features were examined under an Olympus SZ2-ILST stereo microscope. Adult images were captured using a Keyence VHX-6000 imaging system. Genitalia were illustrated using Adobe Illustrator CS6 and Adobe Photoshop CS6. Male genitalia were prepared by boiling in an 8–10% NaOH solution for 1.5–2.5 min, then rinsed with water and stored in glycerol.

Morphological terminology and higher-level classification follow Zhang (1990), Rakitov (1997), and Dietrich (2005). Nomenclature of the new species adheres to the International Code of Zoological Nomenclature (International Commission on Zoological Nomenclature [ICZN] 1999).

Total genomic DNA was extracted from the insect abdomen using the DNeasy^®^ Blood & Tissue Kit (Qiagen, Germany). DNA purity and concentration were assessed using a Nanodrop 2000 spectrophotometer and 1% agarose gel electrophoresis. Extracted DNA was stored at -20 °C.

### ﻿Sequence assembly, annotation, and analysis

The complete mitochondrial genome was sequenced using the Illumina HiSeq 6000 platform (Berry Genomic, Beijing, China), generating 150 bp paired-end reads with an average insert size of 350 bp. Approximately 2 GB of clean data was obtained. Raw sequence data were assembled in Geneious R9 (Kearse et al. 2012), using *C. hamata* (GenBank: MN922303; Xu and Dai 2020) as a reference.

Genome annotation was performed using the MITOS web server (http://mitos2.bioinf.uni-leipzig.de/index.py; Donath et al. 2019). The 13 protein-coding genes (PCGs) were identified using ORF Finder in Geneious Prime with the invertebrate mitochondrial genetic code. The secondary structures of the 22 tRNA genes were predicted using tRNAscan-SE 1.21 and ARWEN 1.2 (Lowe and Eddy 1997; Laslett and Canbäck 2008).

Base composition and relative synonymous codon usage (RSCU) values were calculated using MEGA X (Kumar et al. 2018), which was also used to compute amino acid usage frequency. A graphical map of the mitogenome was generated using the CGView comparison tool (https://proksee.ca; Grant et al. 2023). Strand asymmetry was calculated using the following formulas (Perna and Kocher 1995):

AT skew = (A − T) / (A + T)

GC skew = (G − C) / (G + C)

### ﻿Phylogenetic analyses

To clarify the phylogenetic position of *C. pala* sp. nov., we analyzed its complete mitogenome along with those of 33 additional Deltocephalinae species obtained from GenBank. Two species from the subfamily Iassinae (*Krisna rufimarginata* and *Batracomorphus lateprocessus*) were used as outgroups (Table 1).

**Table 1.** Species, GenBank accession numbers and sources used for this study.

Tribe	Species	GenBank accession number
Acinopterini	*Acinopterus* sp.	OR187394
Acostemmini	*Acostemma* sp.	OR187395
Arrugadini	*Arrugada affinis*	NC085837
Athysanini	*Abrus yunshanensis*	NC065135
	*Abrus daozhenensis*	NC065134
	*Abrus expansivus*	NC045238
	*Paramacrosteles nigromaculatus*	NC045270
Chiasmini	*Nephotettix parvus*	NC073512
	*Zahniserius cylindricus*	NC073513
Cicadulini	*Cicadula* sp.	KX437724
Deltocephalini	*Alobaldia tobae*	KY039116
	*Maiestas dorsalis*	NC036296
Drabescini	*Bhatia longiradiata*	NC085566
	*Drabescoides nuchalis*	NC028154
	*Drabescus ineffectus*	NC050258
	*Roxasellana stellata*	NC050257
Fieberiellini	*Fieberiella septentrionalis*	NC057252
Hecalini	*Hecalus* sp.	OR187399
Iassinae (outgroup)	*Krisna rufimarginata*	NC046068
	*Batracomorphus lateprocessus*	NC045858
Mukariini	*Mukaria splendida*	NC053559
Paralimnini	*Paralaevicephalus gracilipenis*	MK450366
	*Yanocephalus yanonis*	NC036131
Penthimiini	*Chanohirata theae*	NC081953
	*Chanohirata hamata*	MN922303
	*Penthimia melanocephala*	NC051525
	*Chanohirata pala* sp. nov.	PQ615145
Scaphoideini	*Changbaninus pleiospicules*	NC060980
	*Phlogothamnus polymaculatus*	NC060774
	*Mimotettix multispinosus*	NC060773
	*Phlogotettix cyclops*	NC060772
	*Parascaphoidella transversa*	NC060771
	*Scaphoideus maculatus*	NC060770
Selenocephalini	*Selenocephalus* sp.	OR187403

Phylogenetic reconstruction was based on sequences from 13 PCGs and two ribosomal RNA genes (12S and 16S rRNA). These sequences were extracted using Geneious Prime 2019.2.1 (Kearse et al. 2012). Multiple sequence alignment was conducted using MAFFT v7.313 within PhyloSuite v. 1.2.1. Poorly aligned regions and gaps were removed using Gblocks 0.91b (Talavera and Castresana 2007; Katoh et al. 2019; Zhang et al. 2020).

Gene alignments were concatenated using MEGA X (Kumar et al. 2018). Two datasets were prepared: (i) PCG-rRNA dataset: 13 PCGs + 12S + 16S rRNA (12,105 bp), and (ii) CG-only dataset: 13 PCGs (10,875 bp). Phylogenetic trees were constructed using both Maximum Likelihood (ML) and Bayesian Inference (BI) methods based on these datasets (Huelsenbeck and Ronquist 2001; Nguyen et al. 2015).

## ﻿Results

### ﻿Genome organization and nucleotide composition

**Figure 1.**
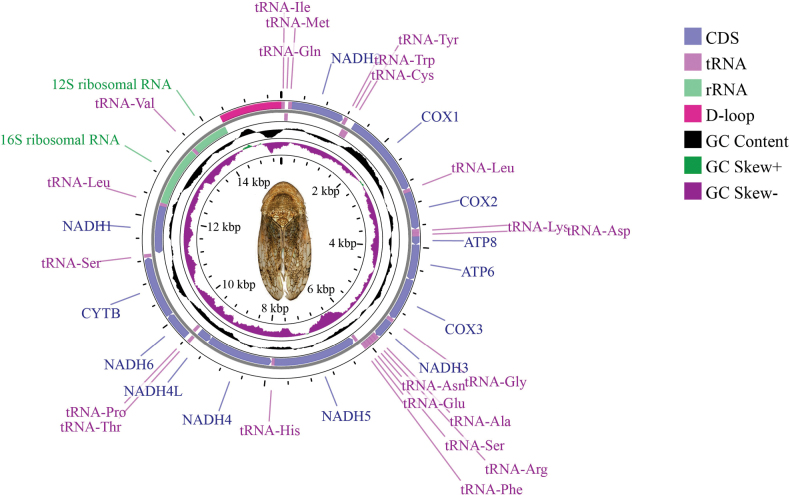
Map of the mitogenome of *C. pala* sp. nov.

The complete mitochondrial genome of *C. pala* sp. nov. (GenBank accession no. PQ615145) is circular, with a total length of 15,433 bp (Fig. 1). It contains 13 protein-coding genes (PCGs), 22 transfer RNA genes (tRNAs), two ribosomal RNA genes (rRNAs), and a control region (CR) rich in A+T nucleotides. Its gene arrangement and composition are consistent with those of other Cicadellidae mitogenomes (Cameron 2014a; Wang et al. 2020; Li et al. 2023; Yang et al. 2023; Lu et al. 2024; Sun et al. 2024). Among the 13 PCGs, four genes (*ND1*, *ND4*, *ND4L*, and *ND5*) are located on the N-strand, while the remaining genes (*ND2*, *ND3*, *ND6*, *COX1*, *COX2*, *CYTB*, *ATP6*, and *ATP8*) are located on the J-strand (Fig. 1), which is typical of Cicadellidae mitogenomes (Wang et al. 2017a, 2017b).

The nucleotide composition of the mitogenome of *C. pala* sp. nov. and its components (whole genome, PCGs, rRNAs, tRNAs, and CR) is shown in Table 3: A (%): 43.2, 43.2, 43.9, 42.4, 42.5; T (%): 33.5, 32.3, 36.7, 37.5, 35.3; G (%): 9.5, 9.8, 7.5, 9.0, 10.2; and C (%): 13.8, 14.7, 11.9, 11.2, 11.6, respectively. The A+T content of the whole mitogenome is 76.7%, and the A+T content in the PCGs, rRNAs, tRNAs, and CR is 75.5%, 80.6%, 79.9%, and 77.8%, respectively (Table 3), indicating a strong A+T bias, similar to other leafhoppers (Wang et al. 2017a, 2017b, 2018). The AT-skew and GC-skew values for the genome, PCGs, rRNAs, tRNAs, and CR are as follows: AT-skew: 0.13, 0.14, 0.09, 0.06, 0.09; and GC-skew: -0.18, -0.20, -0.23, -0.11, -0.06, respectively.

### ﻿PCGs and codon usage

The start and stop codons of the 13 PCGs, as well as the anticodons of the 22 tRNAs, are listed in Table 2. Most PCGs use typical ATN start codons (ATA, ATT, ATC, or ATG) and terminate with either TAR (TAA or TAG) or an incomplete stop codon (T). Notably, *ND1* and *ND4* were reported to end with ATT, and *ND5* initiates with AAC and terminates with a single A, which may be sequencing artifacts or indicative of RNA editing. Among the 13 PCGs, *ND5* is the longest gene (1,660 bp), and *ATP8* is the shortest (148 bp), consistent with other insects (Mao et al. 2017; Wang et al. 2018; Du et al. 2019; Xu and Dai 2021).

**Table 2.** Organization of the mitogenome of *C. pala* sp. nov.

Locus	Position		Size (bp)	Codon			Intergenic nucleotide	Strand
	start	stop		start	stop	anti-codon		
tRNA-I	1	64	64			GAT	0	F
tRNA-Q	129	61	69			TTG	-4	R
tRNA-M	128	195	68			CAT	-2	F
*ND2*	196	1164	969	ATA	TAA		0	F
tRNA-W	1173	1239	67			TCA	8	F
tRNA-C	1298	1231	68			GCA	-9	R
tRNA-Y	1366	1301	66			GTA	2	R
*COX1*	1381	2919	1539	ATG	TAA		14	F
tRNA-L1	2919	2982	64			TAA	-1	F
*COX2*	2983	3663	681	ATA	TAA		0	F
tRNA-K	3668	3736	69			CTT	4	F
tRNA-D	3736	3800	65			GTC	-1	F
*ATP8*	3801	3948	148	ATT	T-		0	F
*ATP6*	3950	4594	645	ATA	TAA		1	F
*COX3*	4597	5374	778	ATG	T-		2	F
tRNA-G	5375	5437	63			TCC	0	F
*ND3*	5444	5791	348	ATA	TAG		6	F
tRNA-A	5789	5855	67			TGC	-3	F
tRNA-R	5854	5919	66			TCG	-2	F
tRNA-N	5917	5984	68			GTT	-3	F
tRNA-S1	5983	6049	67			GCT	-2	F
tRNA-E	6049	6116	68			TTC	-1	F
tRNA-F	6178	6116	67			GAA	-1	R
*ND5*	7851	6192	1660	AAC	A-		13	R
tRNA-H	7911	7851	61			GTG	-1	R
*ND4*	9232	7916	1317	TAA	ATT		4	R
*ND4L*	9495	9234	262	TAA	T-		1	R
tRNA-T	9497	9560	64			TGT	1	F
tRNA-P	9625	9560	66			TGG	-1	R
*ND6*	9628	10120	493	ATT	T-		2	F
*CYTB*	10121	11245	1125	ATC	TAA		0	F
tRNA-S2	11252	11314	63			TGA	6	F
*ND1*	12246	11308	939	TAA	ATT		-7	R
tRNA-L2	12311	12246	66			TAG	-1	R
*16S-rRNA*	13495	12315	1181				3	R
tRNA-V	13569	13503	67			TAC	7	R
*12S-rRNA*	14299	13569	731				-1	R
CR	14300	15433	1134				0	

**Table 3.** Nucleotide composition of the *C. pala* sp. nov. mitogenome.

	Length (bp)	A%	C%	T%	G%	A+T%	AT-skew%	GC-skew%
Genome	15433	43.2	13.8	33.5	9.5	76.7	0.13	-0.18
PCGs	10904	43.2	14.7	32.3	9.8	75.5	0.14	-0.20
rRNA	1912	43.9	11.9	36.7	7.5	80.6	0.09	-0.23
tRNA	1449	42.4	11.2	37.5	9.0	79.9	0.06	-0.11
CR	1134	42.5	11.6	35.3	10.2	77.8	0.09	-0.06

**Figure 2.**
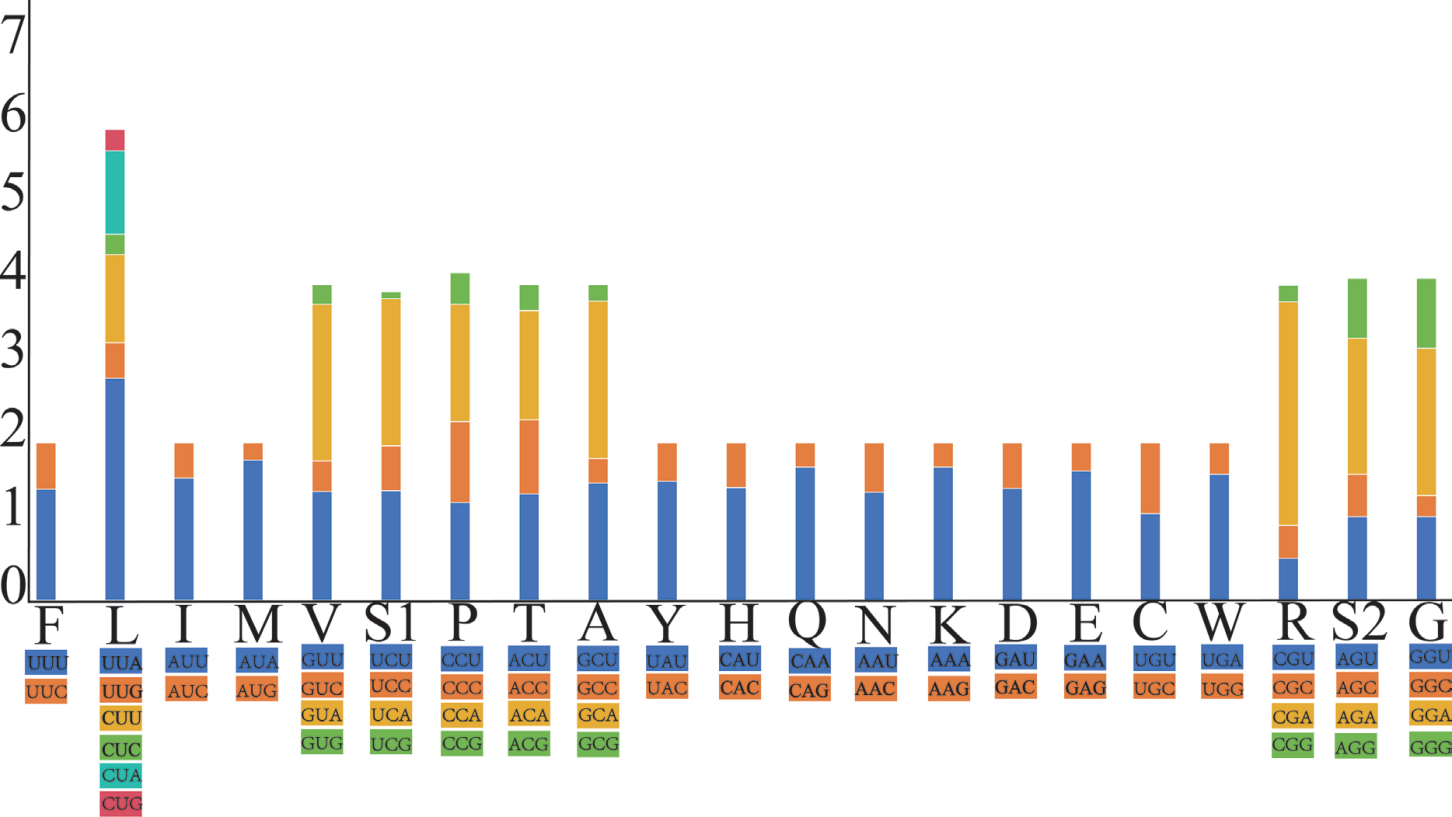
RSCU in the mitogenome of *C. pala* sp. nov.; the stop codon is not included.

**Figure 3.**
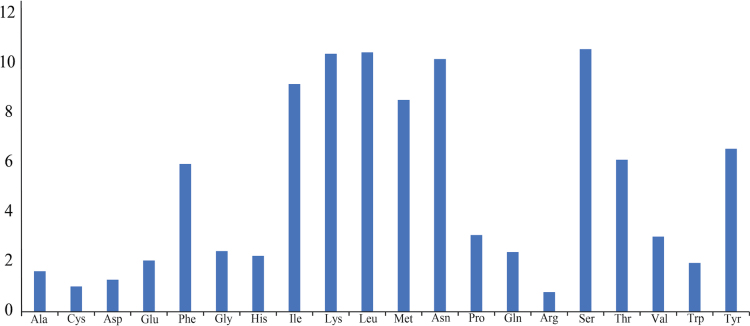
Overall amino acid usage frequency in the mitogenome of *C. pala* sp. nov.

Figs 2, 3 summarize the relative synonymous codon usage (RSCU) and amino acid frequency. The most frequently used codon is CGA (Arg), followed by UUA (Leu), whereas the least used is UCG (Ser1). The most frequently encoded amino acids are Ser, Leu, Lys, Asn, and Ile. The prevalent use of codons composed entirely of A or T reflects the high A+T content of the genome.

### ﻿tRNAs, rRNAs, and CR

**Figure 4.**
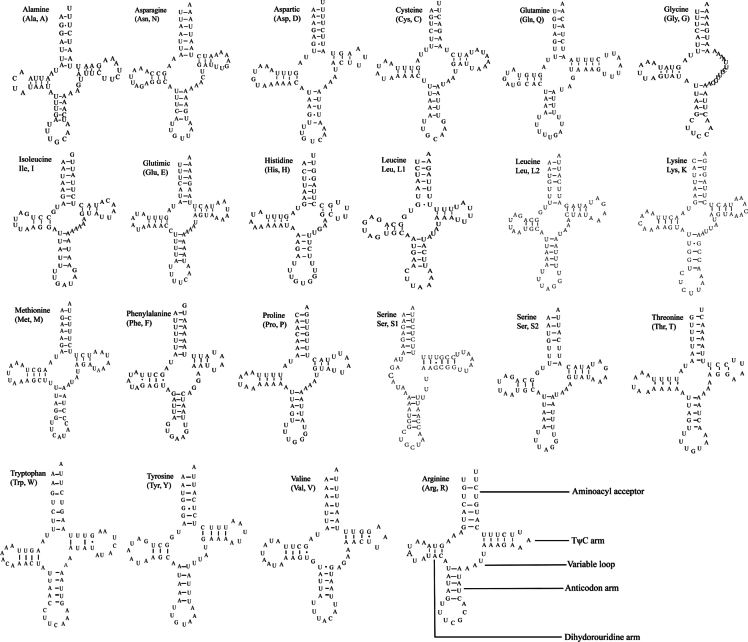
Predicted secondary structures of 22 tRNAs.

All 22 tRNAs were predicted using tRNAscan-SE 1.21, ARWEN 1.2, and MITOS2. Most tRNAs exhibit the typical cloverleaf secondary structure, except for *trnS1*, which lacks the dihydrouridine (DHU) arm and instead forms a simple loop, with is a common feature in many insects (Sheffield et al. 2008; Cao and Du 2014; Wang et al. 2014; Zhang et al. 2014; Wang et al. 2017a, b). Additionally, trnG lacks the TΨC arm (Fig. 4). The lengths of the tRNAs range from 61 bp (tRNA-His) to 69 bp (tRNA-Gln) (Table 2). Several base mismatches were observed in tRNA secondary structures (Table 4), including: A-A mismatches (e.g., in trnE), U-U mismatches (e.g., in *trnQ*, *trnL1*, *trnL2*, *trnW*, and *trnR*), and G-U mismatches, which were most frequent, observed in 13 tRNAs (*trnA*, *trnD*, *trnQ*, *trnG*, *trnH*, *trnL1*, *trnK*, *trnF*, *trnP*, *trnS1*, *trnW*, *trnY*, *trnV*). These findings suggest that further research on tRNA secondary structures is warranted.

**Table 4.** Total numbers of different types of base mismatches in tRNAs.

Species	G-U	U-U	A-A
*C. pala* sp. nov.	*trnA* (1)		
	*TrnD* (2)		
	*TrnQ* (1)		
	*TrnG* (2)		
	*TrnH* (2)	*trnQ* (1)	
	*TrnL1* (1)	*TrnL1* (1)	
	*TrnK* (2)	*TrnL2* (2)	*trnE* (1)
	*TrnF* (2)	*trnW* (1)	
	*TrnP* (1)	*trnR* (2)	
	*TrnS1* (1)		
	*TrnW* (1)		
	*TrnY* (1)		
	*TrnV* (3)		

As in other insect mitogenomes, *C. pala* sp. nov. contains two rRNA genes: *rrnL* (1,181 bp) and *rrnS* (731 bp) (Table 2). Their locations are conserved: *rrnL* lies between *trnL2* and *trnV*, and *rrnS* lies between *trnV* and the control region (CR) (Table 2, Fig. 1). The CR (also called the A+T-rich region) is the longest non-coding segment and varies greatly among species in both length and sequence composition, which is a key factor in mitogenome diversity among leafhoppers (Wang et al. 2020). In *C. pala* sp. nov., the CR is 1,134 bp in length, located between rrnS and trnI, with an A+T content of 77.8% (Tables 2, 3).

### ﻿Phylogenetic analysis

The phylogenetic relationships of Deltocephalinae were inferred using 34 species based on two datasets: (i) 13 protein-coding genes (PCGs) and two ribosomal RNA genes (12S and 16S rRNA), comprising 12,105 nucleotides (PCG+rRNA); and (ii) PCGs only, comprising 10,875 nucleotides. Four phylogenetic trees were generated using Bayesian Inference (BI) and Maximum Likelihood (ML) methods (BI-PCGs, BI-PCG+rRNA, ML-PCGs, ML-PCG+rRNA), all of which yielded similar topologies.

**Figure 5.**
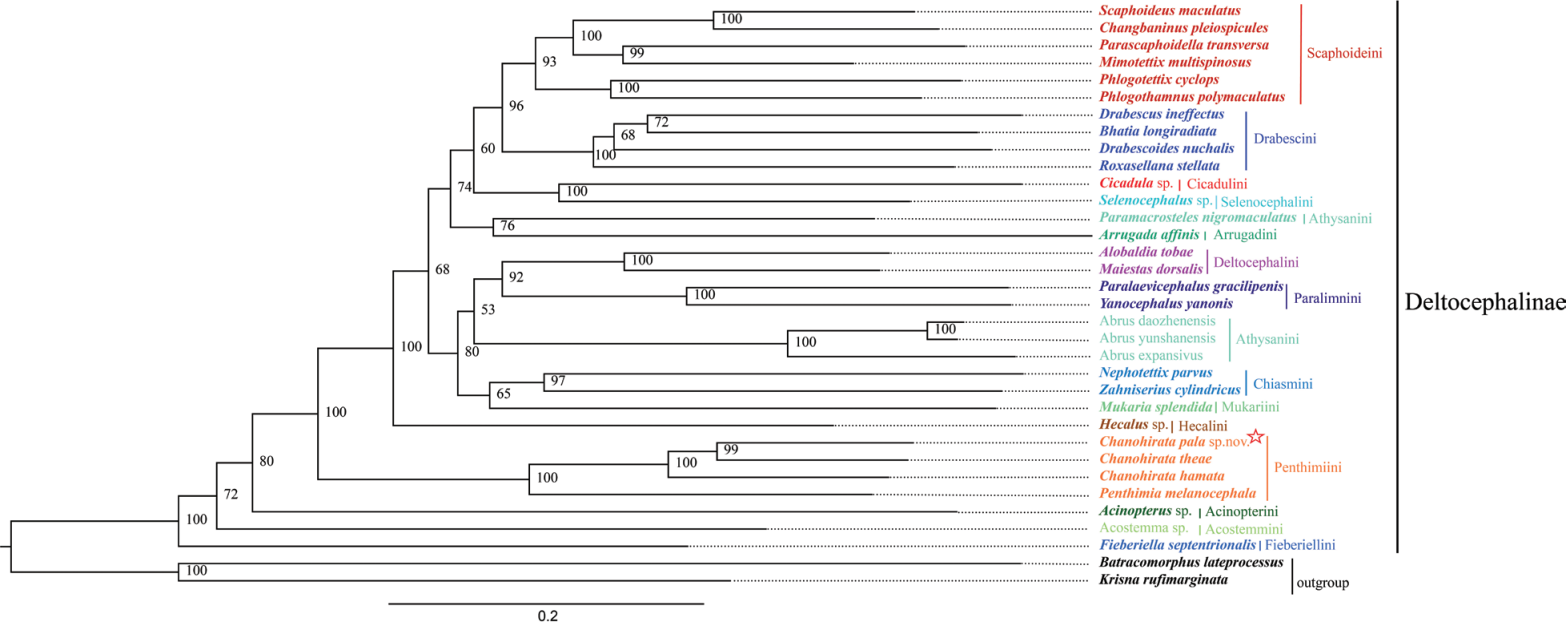
ML tree resulting from the analysis of 13 PCGs of mitogenomes in the Deltocephalinae.

**Figure 6.**
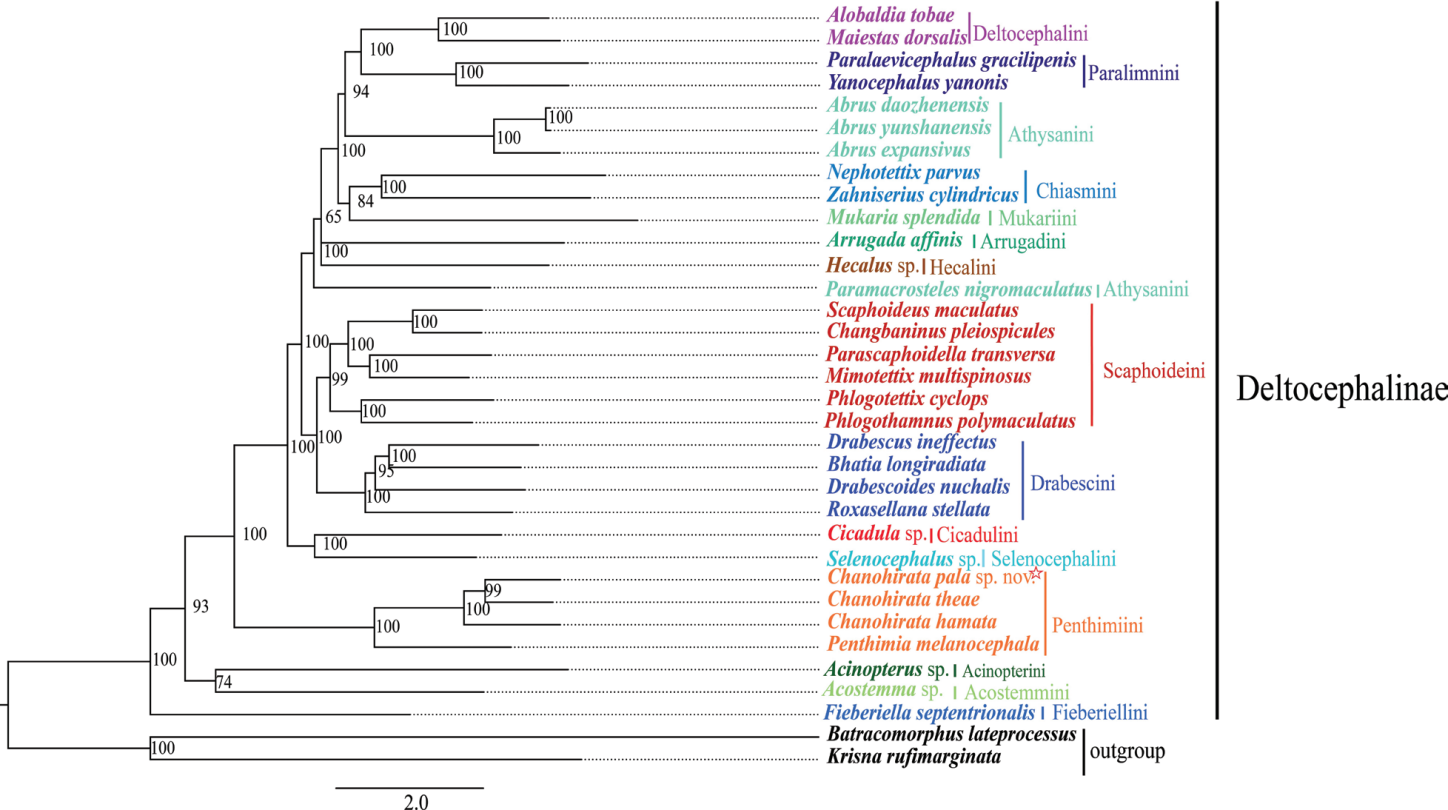
BI tree resulting from the analysis of 13 PCGs of mitogenomes in the Deltocephalinae.

Previous studies have examined the phylogenetic relationships within Deltocephalinae using either morphological or molecular data (Zahniser and Dietrich 2008, 2010, 2013; Du et al. 2017a, 2017b; Hassan et al. 2023). In our study, mitogenomic data produced well-resolved trees supporting several established relationships. The results indicate that Penthimiini occupies a basal position in the phylogenetic tree (Figs 5, 6; Suppl. material 1: figs S1, S2), which is consistent with previous findings (Zahniser and Dietrich 2013; Wu et al. 2021). *C. pala* sp. nov. clusters with other Deltocephalinae species and specifically with *C. theae* and *C. hamata* within Penthimiini.

Within Deltocephalinae, Scaphoideini forms a distinct clade with Drabescini, with both lineages recovered as sister groups (Figs 5, 6; Suppl. material 1: figs S1, S2), corroborating previous findings by Zahniser and Dietrich (2013), Xu et al. (2020b), and Lu et al. (2023). Stable sister-group relationships were also observed between Deltocephalini and Paralimnini, and between Selenocephalini and Cicadulini. However, the phylogenetic placement of Athysanini remained unresolved and unstable across datasets, echoing earlier studies (Wu et al. 2021; Hassan et al. 2023). In addition, the monophyly of Chiasmini, Deltocephalini, Drabescini, Paralimnini, Penthimiini, and Scaphoideini was strongly supported in all analyses (Figs 5, 6; Suppl. material 1: figs S1, S2).

### ﻿Taxonomy

#### ﻿Genus Chanohirata

Taxon classificationAnimaliaHemipteraCicadellidae

Hayashi & Machida 1996

F4265F76-348C-59F3-9C8A-F63024117990

Chanohirata Hayashi & Machida, 1996: 69–70; Wang & Zhang, 2022: (3): 432–441; Wang, Dietrich, & Zhang, 2024: 49(1): 173–192.

##### Type species.

*Penthimia theae* Matsumura, 1912.

##### Description.

Reticulate color pattern on head, pronotum, scutellum, forewings differ from other genera of Penthimiini. Distinct boundary between crown and face. Scutellum with rounded apical margin, prominent transverse striations. Females: terminal abdominal segment (telson) conical, longer than wide, apical half with setae. Hind femur with setal formula 2 + 2 + 1. Male pygofer quadrate, without latero-apical process. Connective Y- or T-shaped. Forewings subhyaline, with supernumerary costal cross-veins, numerous false veins composed of pigmented lines. Male genitalia short, curved dorsally, flattened lateral margins, genital opening apical.

##### Remarks.

*Chanohirata* resembles *Penthimia* in general body structure but differs by its reticulate dorsal color pattern and the clearly defined boundary between the crown and the face. It also shows similarities to *Uzelina* Melichar (known from sub-Saharan Africa and Sri Lanka) in male genitalia, however, *Uzelina* has a spatulate head in profile and lacks the reticulate markings seen on the vertex of *Chanohirata*. Prior to this study, the genus comprised 15 described species distributed globally. Here, we describe a new species belonging to this genus.

##### Distribution.

China (Anhui, Fujian, Guangdong, Guangxi, Guizhou, Hainan, Henan, Hunan, Jiangxi, Shaanxi, Taiwan, Tibet, Yunnan, Zhejiang); Japan (Fig. 9).

###### ﻿Checklist of the genus *Chanohirata*

***Chanohirata theae* (Matsumura, 1912)**

*Penthimia theae* Matsumura, 1912: 50.

*Chanohirata theae* (Matsumura, 1912): Hayashi and Machida 1996: 70–72.

*Penthimia testacea* Kuoh, 1991: 206–207.

*Reticuluma testacea* (Kuoh, 1991): Fu and Zhang 2015: 257.

**Distribution.** China (Anhui, Fujian, Guangdong, Guangxi, Guizhou, Henan, а, Jiangxi, Shaanxi, Taiwan, Yunnan, Zhejiang); Japan (Fig. 9).

***Chanohirata citrana* (Cheng & Li, 2005)**

*Reticuluma citrana* Cheng & Li, 2005: 381.

*Chanohirata citrana* (Cheng & Li, 2005): Wang, Dietrich and Zhang 2023: 183.

**Distribution.** China (Guizhou) (Fig. 9).

***Chanohirata spinata* (Cheng & Li, 2005)**

*Reticuluma spinata* Cheng & Li, 2005: 381.

*Chanohirata spinata* (Cheng & Li 2005): Wang and Zhang 2022: 434.

**Distribution.** China (Guizhou) (Fig. 9).

***Chanohirata lini* (Cheng & Li, 2005)**

*Reticuluma lini* Cheng & Li, 2005: 380.

*Chanohirata lini* (Cheng & Li, 2005): Wang and Zhang 2022: 434.

**Distribution.** China (Guizhou) (Fig. 9).

***Chanohirata dactyla* (Fu & Zhang, 2015)**

*Reticuluma dactyla* Fu & Zhang, 2015: 254.

*Chanohirata dactyla* (Fu & Zhang, 2015): Wang and Zhang 2022: 434.

**Distribution.** China (Guizhou, Shaanxi) (Fig. 9).

***Chanohirata eurya* (Fu & Zhang, 2015)**

*Reticuluma eurya* Fu & Zhang, 2015: 256.

*Chanohirata eyrya* (Fu & Zhang, 2015): Wang and Zhang 2022: 434.

**Distribution.** China (Tibet, Yunnan) (Fig. 9).

***Chanohirata hamata* (Wang & Zhang, 2019)**

*Reticuluma hamata* Wang & Zhang, 2019: 291.

*Chanohirata hamata* (Wang & Zhang, 2019): Wang and Zhang 2022: 434.

**Distribution.** China (Yunnan) (Fig. 9).

***Chanohirata lageniformia* (Wang & Zhang, 2019)**

*Reticuluma lageniformia* Wang & Zhang, 2019: 291.

*Chanohirata lageniformia* (Wang & Zhang, 2019): Wang and Zhang 2022: 434.

**Distribution.** China (Guangxi, Hainan, Yunnan) (Fig. 9).

***Chanohirata bipennata* (Xu, Yu, Dai & Yang, 2020)**

*Reticuluma bipennata* Xu, Yu, Dai & Yang, 2020: 179.

*Chanohirata bipennata* (Xu, Yu, Dai & Yang, 2020): Wang and Zhang 2022: 434.

**Distribution.** China (Yunnan) (Fig. 9).

***Chanohirata yunnana* Wang & Zhang 2022**

*Chanohirata yunnana* Wang & Zhang, 2022: 438.

**Distribution.** China (Yunnan) (Fig. 9).

***Chanohirata minima* Wang & Zhang 2022**

*Chanohirata minima* Wang & Zhang, 2022: 435.

**Distribution.** China (Yunnan) (Fig. 9).

***Chanohirata plania* Wang & Zhang 2022**

*Chanohirata plania* Wang & Zhang, 2022: 436.

**Distribution.** China (Tibet) (Fig. 9).

***Chanohirata cornicula* Wang & Zhang, 2023**

*Chanohirata cornicula* Wang & Zhang in Wang, Dietrich and Zhang 2023: 186.

**Distribution.** China (Yunnan) (Fig. 9).

***Chanohirata elongata* Wang & Zhang, 2023**

*Chanohirata elongata* Wang & Zhang in Wang, Dietrich and Zhang 2023: 187.

**Distribution.** China (Yunnan) (Fig. 9).

***Chanohirata serrata* Wang & Zhang, 2023**

*Chanohirata serrata* Wang & Zhang in Wang, Dietrich and Zhang 2023: 187.

**Distribution.** China (Yunnan) (Fig. 9).

***Chanohirata pala* sp. nov.**

**Distribution.** China (Yunnan) (Fig. 9).

### ﻿Key to species of *Chanohirata*

(males, Fig. 10A–P)

**Table:** 

1	Connective approximately T-shaped	**2**
–	Connective approximately Y-shaped	**5**
2	Pygofer side with process at apex on lateral margin	***C. citrana***
–	Pygofer side without process at apex on lateral margin	**3**
3	Aedeagal shaft broadest near apex in ventral view	***C. eurya***
–	Aedeagal shaft broadest near base in ventral view	**4**
4	Apex of aedeagus with small teeth on each side	***C. yunnana***
–	Apex of aedeagus with two spines	***C. spinata***
5	Aedeagus shaft with process	**6**
–	Aedeagus shaft is smooth, without process	**15**
6	Aedeagus with one pair of processes	**7**
–	Aedeagus with two pairs of processes	**11**
7	Process of aedeagus near base	**8**
–	Process of aedeagus near apex	**12**
8	Process of aedeagus longer, approx. equal to aedeagus length	**9**
–	Process of aedeagus shorter, approx. 1/4 aedeagus length	**10**
9	Basal processes of aedeagus wing-shaped	***C. bipennata***
–	Basal processes of aedeagus slender with bilobed apices	***C. elongata***
10	Basal processes of aedeagus with tuberculate	***C. lageniformia***
–	Basal processes of aedeagus with one pair of plate-like processes	***C. hamata***
11	Aedeagus with triangular lateral subapical process	***C. minima***
–	Aedeagal shaft with lateral margins serrated	***C. theae***
12	The margins of processes of aedeagus with finely serrated	**13**
–	The margins of processes of aedeagus smooth	**14**
13	Aedeagus with a pair of plate-like processes	***C. serrata***
–	Aedeagus with triangular lateral processes apically	***C. plania***
14	Aedeagus with a pair of hook-like processes	***C. lini***
–	Aedeagus with paired lateral flanges of horned process	***C. cornicula***
15	Base of aedeagus distinctly broad, shovel-shaped (Fig. 10P)	***C. pala* sp. nov.**
–	Base of aedeagus not broad, digitiform	***C. dactyla***

#### ﻿ Chanohirata pala

Taxon classificationAnimaliaHemipteraCicadellidae

Sun, Xu & Dai, sp. nov.

DDB8276B-1AD5-5F45-B0B3-E002CDD71EB6

https://zoobank.org/99625229-5FA8-4FC1-8920-192A59056F22

Figs 7A–D, 8A–E

##### Type material.

***Holotype***: • ♂ (GUGC), China, Guizhou Province, Yanhe County, Mayang River National Nature Reserve, 15 August 2014, coll. Wu Yunfei. ***Paratypes***: • 1♂ (GUGC), China, Yunnan Province, Yingjiang County, Copper Wall Pass, 30 May 2019, coll. Zuo Qin. • 1♂ (GUGC), China, Guangxi Province, Longzhou County, Nonggang Town, 10 May 2021, coll. Lu Jikai.

##### Measurement.

Body length (including tegmen): male 4.0–4.2 mm.

##### Coloration.

**Figure 7.**
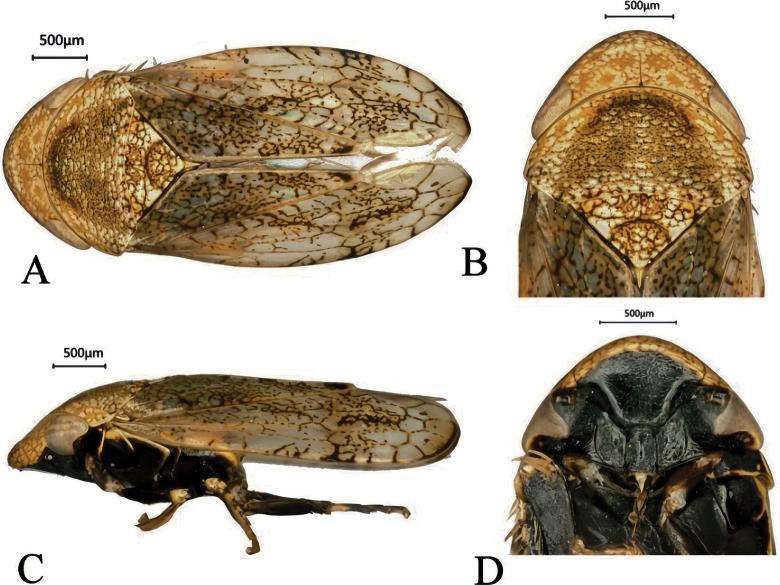
*C. pala* sp. nov. A. Habitus, dorsal view; B. Head and pronotum, dorsal view; C. Habitus, lateral view; D. Face.

Body yellowish brown. Vertex, pronotum, and scutellum marked with black reticulations (Fig. 7A). Ocelli brownish; compound eyes blackish gray (Fig. 7C). Face black (Fig. 7D); pronotum mostly yellow (Fig. 7B). Forewing subhyaline, grayish white, with yellowish brown reticulations; claw folds and veins light brown; apical margin brown (Fig. 7A).

##### Morphology.

Vertex with anterior margin prominent, slightly thickened, arcuate, bearing wavy lines; posterior margin broadly concave. Vertex length approximately one-third of its width (Fig. 7B). Ocelli positioned anterior to the compound eyes and close to their anterior margins (Fig. 7B, C). Face and clypeus flat; pre-clypeus slightly raised. Lateral plates of the labium broad (Fig. 7D). Pronotum about half as long as wide. Scutellum broader than long, triangular, with a deep, arcuate transverse impression (Fig. 7B).

##### Male genitalia.

**Figure 8.**
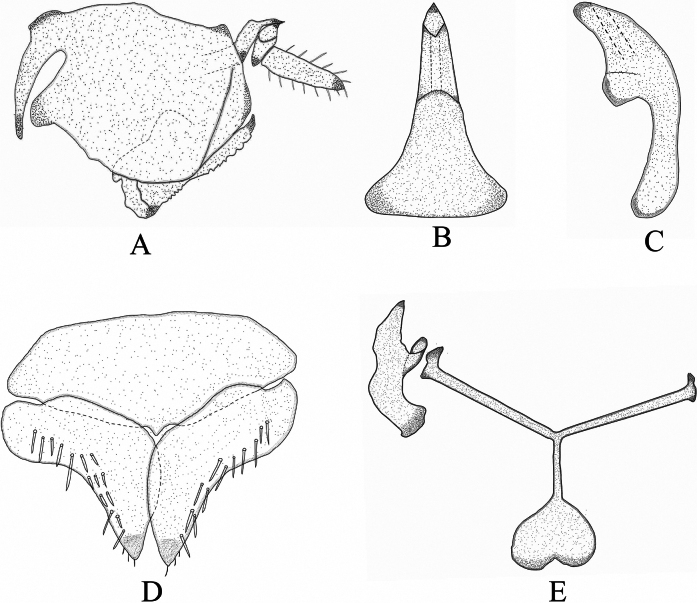
*C. pala* sp. nov. A. Male genital capsule, lateral view; B. Aedeagus, dorsal view; C. aedeagus, lateral view; D. Subgenital plate and genital valve, ventral view; E. Style and connective, ventral view.

Pygofer longer than tall, posterolateral margin bearing both long and short macrosetae (Fig. 8A). Subgenital plate bluntly rounded at apex, ventrally with several scattered macrosetae; basal lobe nearly semicircular. Subgenital plate nearly quadrangular, with coarse, short setae along the outer margin and apex (Fig. 8D). Connective approximately Y-shaped, with arms longer than the stem (Fig. 8E). Lateral processes of the style short, compact, with pointed tips (Fig. 8E). Aedeagus robust, with a broad base and shovel-shaped overall form (Fig. 8B).

##### Etymology.

The specific epithet *pala* is derived from Latin, meaning shovel, referring to the broad, shovel-shaped base of the aedeagus.

##### Remarks.

This species is similar to *C. dactyla* but can be distinguished by the shape of the aedeagus.

## ﻿Discussion

The primary goal of this study was to describe a new species, *C. pala* sp. nov., collected from Yunnan, China, investigate the structural characteristics of its mitochondrial genome and phylogenetic placement within the tribe Deltocephalinae. While previous studies have explored the phylogeny of Deltocephalinae using morphological and molecular data (Zahniser and Dietrich 2013; Wang and Xing 2019; Luo et al. 2021; Wu et al. 2021; Yang and Dai 2021; Cao et al. 2022; Lu et al. 2023; Hassan et al. 2023; Wang et al. 2024a), but focused research on the tribe Penthimiini remains limited. To date, only one phylogenetic reconstruction of the genus *Chanohirata* has been conducted (Wang et al. 2024a), and only four complete mitochondrial genomes from Penthimiini are available in GenBank.

This study contributes a new complete mitochondrial genome for *C. pala* sp. nov., significantly expanding the mitogenomic resources for the tribe. Our phylogenetic analysis based on mitochondrial protein-coding and rRNA genes successfully recovered several tribes within Deltocephalinae as monophyletic, including Chiasmini, Deltocephalini, Drabescini, Paralimnini, Penthimiini, and Scaphoideini, while Athysanini was not supported as monophyletic, these results are consistent with earlier findings by Hassan et al. (2023). Phylogenetic analyses based on mitochondrial genes confirmed that *C. pala* sp. nov. clusters with other Deltocephalinae species and belongs to the genus *Chanohirata*, forming a clade with *C. theae* and *C. hamata*; therefore, we speculate that the origin of *C. pala* sp. nov. was in southern China during the late Eocene (Evans 1972; Cao et al. 2022; Wang et al. 2024a). However, the monophyly and interrelationships of several other tribes, such as Athysanini, Acinopterini, Acostemmini, Arrugadini, Fieberiellini, Hecalini, Mukariini, and Selenocephalini, remain unresolved due to a lack of comprehensive mitogenomic data. Nonetheless, the major research advances of this study include the formal description of a new species with detailed morphological and molecular data, the first integrated mitogenomic analysis of *C. pala* sp. nov., and support for the monophyly of several key tribes within Deltocephalinae based on expanded genomic data. We believe that innovative aspects include the use of complete mitogenomic sequencing to overcome the limitations of morphology-based identification in a morphologically conserved genus. Future directions should prioritize broader taxon sampling and the generation of more mitogenomic datasets for Deltocephalinae. Increasing the number of complete mitochondrial genomes will enhance phylogenetic resolution and enable a more comprehensive understanding of evolutionary patterns within this diverse subfamily. Supplementing existing molecular data is thus critical to clarifying the deep relationships among leafhopper lineages.

**Figure 9.**
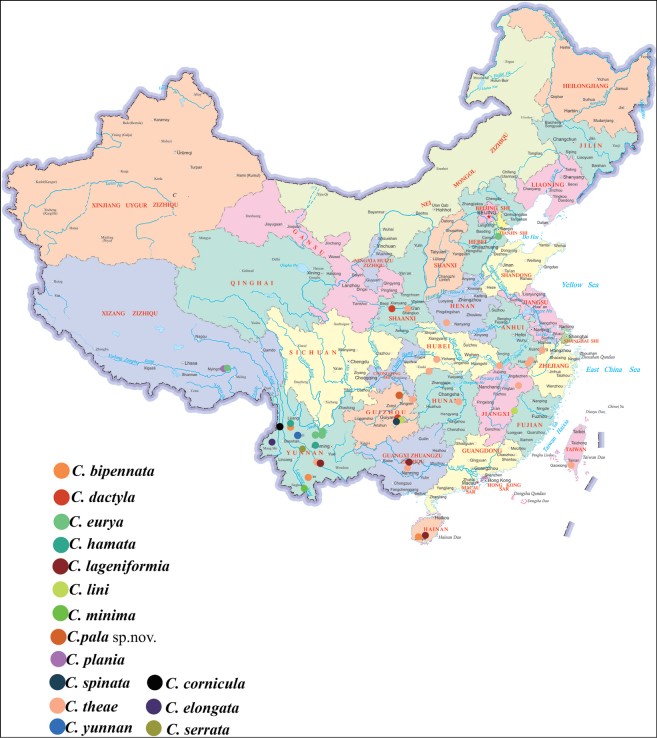
Distribution map of *Chanohirata* species from China.

**Figure 10.**
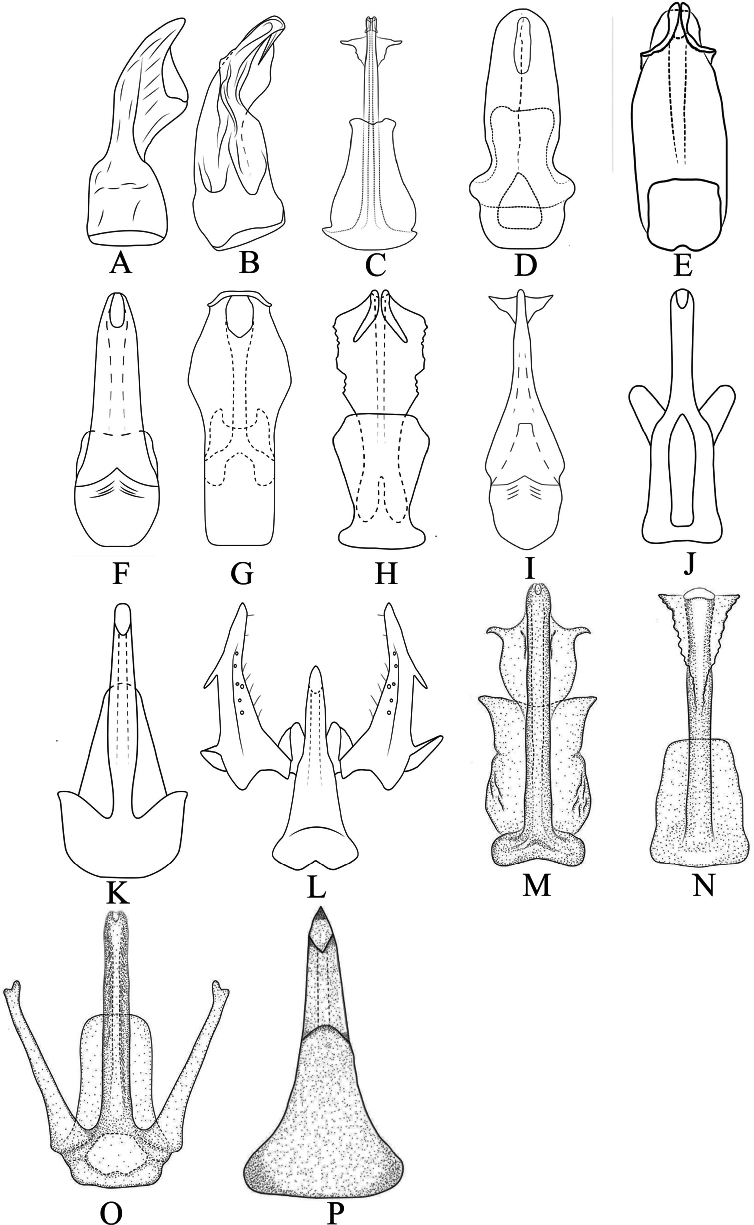
Male aedeagus of *Chanohirata*. A. *C. citrana* lateral view (Cheng & Li, 2005); B. *C. spinata* lateral view (Cheng & Li, 2005); C. *C. minima* dorsal view (Wang & Zhang, 2022) ; D. *C. yunnana* ventral view (Wang & Zhang, 2022); E. *C. plania* dorsal view (Wang & Zhang, 2022); F. *C. dactyla* dorsal view (Fu & Zhang, 2015); G. *C. eurya* dorsal view (Fu & Zhang, 2015); H. *C. theae* ventral view (Fu & Zhang, 2015); I. *C. lini* dorsal view (Cheng & Li, 2005); J. *C. lageniformia* posterior view (Wang & Zhang, 2019); K. *C. hamata* posterior view (Wang & Zhang, 2019); L. *C. bipennata* dorsal view (Xu et al. 2020a); M. *C. cornicula* ventral view (Wang & Zhang, 2024a); N. *C. serrata* ventral view (Wang & Zhang, 2024a); O. *C. elongata* ventral view (Wang & Zhang, 2024a); P. *C. pala.* sp. nov. dorsal view.

In addition, we examined several mitochondrial features of *C. pala* sp. nov., including genome organization, base composition, relative synonymous codon usage, amino acid usage, and tRNA secondary structures. These characteristics were largely consistent with patterns observed in previous leafhopper mitogenome studies (Wu et al. 2016; Yu et al. 2017; Wang et al. 2017a, 2017b, 2019, Wang and Xing 2019; Mao et al. 2017; Du et al. 2017a, 2019; Lu et al. 2024; Sun et al. 2024). Currently, the genus *Chanohirata* comprises 16 known species, which can be distinguished based on differences in aedeagal morphology (Fig. 10). Most species are distributed in southwestern China, with a few extending into adjacent regions (Fig. 9). This study underscores the importance of combining molecular and morphological approaches in taxonomic research. The integrative framework presented here offers new insights into the systematics and evolutionary history of *Chanohirata*, setting the foundation for further phylogenetic and biogeographic studies within the Penthimiini and broader Deltocephalinae.

## ﻿Conclusions

In this study, we described and conducted a phylogenetic analysis of a new species, *C. pala* sp. nov., from Yunnan Province, southern China. The results highlight the significance of integrating molecular and morphological data in taxonomic studies. Phylogenetic analyses based on mitochondrial genes confirmed that *C. pala* sp. nov. clusters with other Deltocephalinae species and belongs to the tribe Penthimiini, forming a clade with *C. theae* and *C. hamata*. We also sequenced and assembled the complete mitogenome of *C. pala* sp. nov., which is 15,433 bp in length and available under GenBank accession number PQ615145. Mitochondrial features such as genome organization, base composition, codon usage, amino acid frequency, and tRNA secondary structures were characterized and found to be consistent with those of related species. However, the monophyly and interrelationships of some Deltocephalinae tribes remain unresolved due to insufficient mitogenome data. Therefore, expanding molecular datasets is critical for future phylogenetic studies and for refining the taxonomy and evolutionary understanding of Deltocephalinae.

## Supplementary Material

XML Treatment for Chanohirata

XML Treatment for Chanohirata pala

## ﻿Additional information

### Use of AI

No use of AI was reported.

### Author ORCIDs

Mingming Sun https://orcid.org/0009-0000-3702-109X

Guy Smagghe https://orcid.org/0000-0001-8334-3313

Renhuai Dai https://orcid.org/0009-0003-2118-9316

### Data availability

All of the data that support the findings of this study are available in the main text or Supplementary Information. The following information was supplied regarding the deposition of DNA sequences: *Chanohirata pala* sp. nov.: PQ615145.

## Supplementary materials

Supplementary material 1 Phylogenetic Tree Data type docx Explanation note ML and BI tree resulting from the analysis of PCGs-12S16S of mitogenomes in the Deltocephalinae. https://binary.pensoft.net/file/1463955This dataset is made available under the Open Database License (http://opendatacommons.org/licenses/odbl/1.0/). The Open Database License (ODbL) is a license agreement intended to allow users to freely share, modify, and use this Dataset while maintaining this same freedom for others, provided that the original source and author(s) are credited.Mingming Sun, Tielong Xu, Guy Smagghe, Renhuai Dai, Xianyi Wang, Jiajia Wang
