# Supplementary material for: ﻿A new species of Chanohirata (Hemiptera, Cicadellidae, Deltocephalinae, Penthimiini) from southern China with its complete genome
Source: Zookeys. 2025 Nov 12;1259:309–33. doi: 10.3897/zookeys.1259.163605 (PMC12631135; doi:10.3897/zookeys.1259.163605)
Supplement: Supplementary material 1 — Phylogenetic Tree [file zookeys-1259-309_article-163605__-s001.docx]

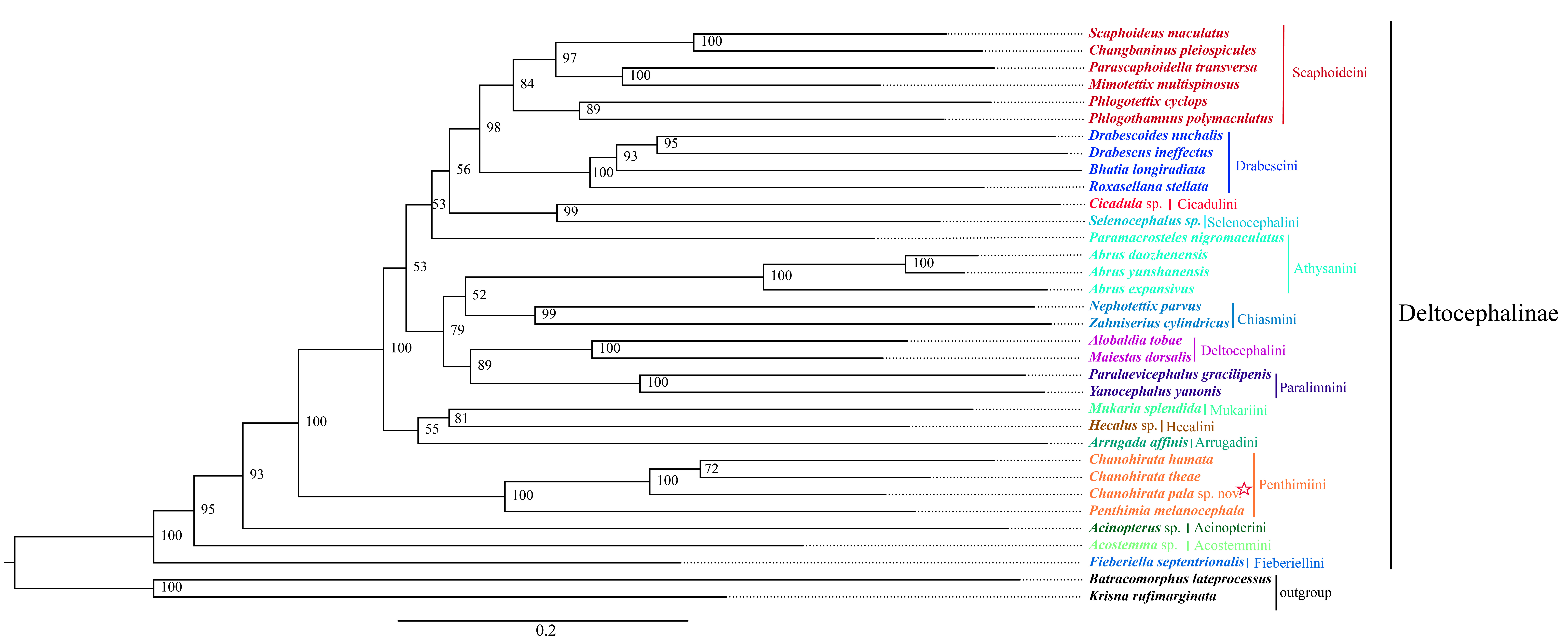


**Figure S1. ML tree resulting from the analysis of PCGs**-**12S16S of mitogenomes in the Deltocephalinae.**

**
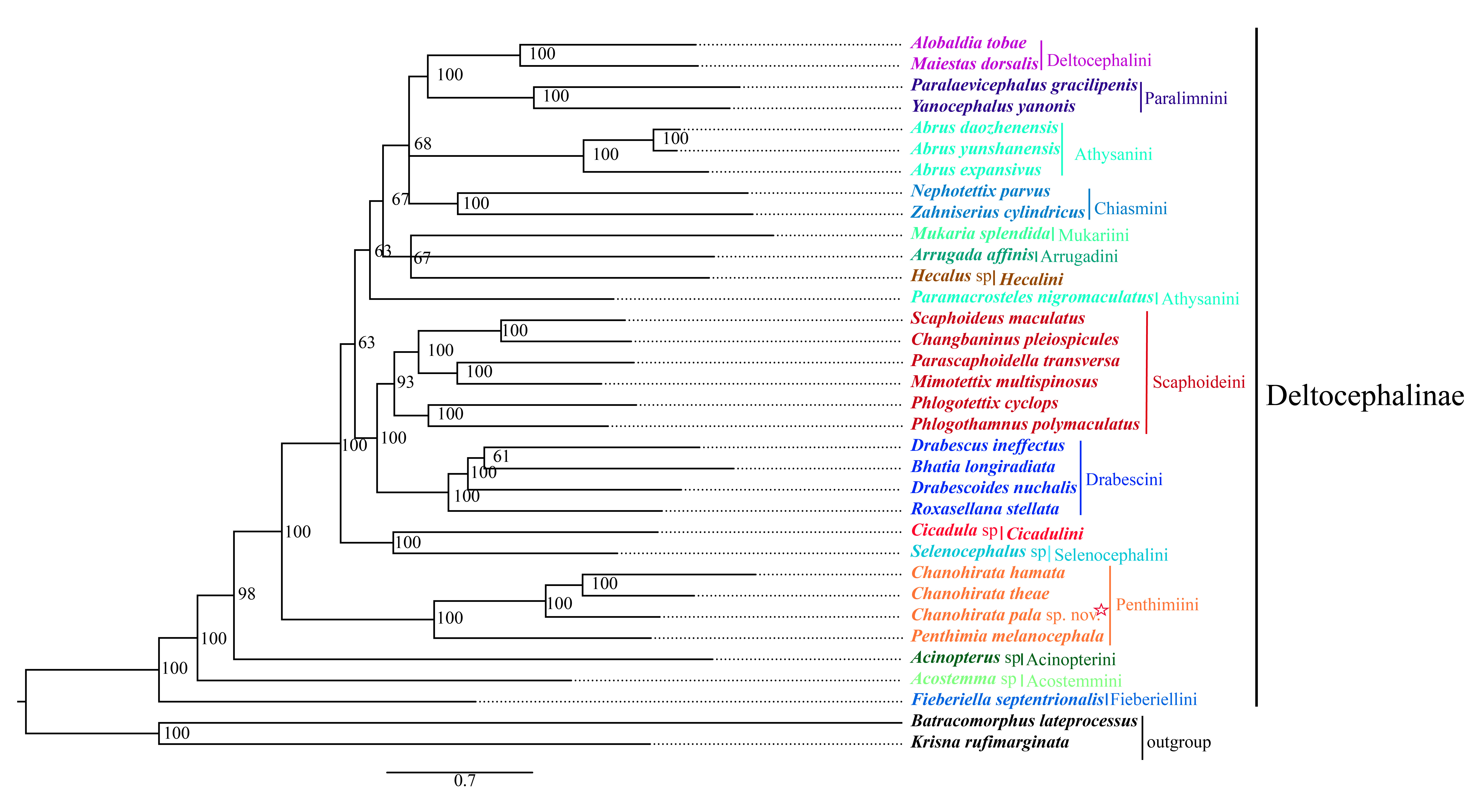
**

**Figure S2. BI tree resulting from the analysis of PCGs**-**12S16S of mitogenomes in the Deltocephalinae.**
